# Optimized Method for the Synthesis of Alkyne-Modified 2′-Deoxynucleoside Triphosphates

**DOI:** 10.3390/molecules29194747

**Published:** 2024-10-08

**Authors:** Viktoriya E. Kuznetsova, Valeriy E. Shershov, Georgiy F. Shtylev, Ivan Yu. Shishkin, Veronika I. Butvilovskaya, Andrey A. Stomakhin, Irina V. Grechishnikova, Olga A. Zasedateleva, Alexander V. Chudinov

**Affiliations:** Engelhardt Institute of Molecular Biology, Russian Academy of Sciences, 119991 Moscow, Russia; shershov@list.ru (V.E.S.); gosha100799@mail.ru (G.F.S.); shishkin.iv2017@yandex.ru (I.Y.S.); v.butvilovskaya@gmail.com (V.I.B.); lana3@mail.ru (O.A.Z.); chud@eimb.ru (A.V.C.)

**Keywords:** modified nucleoside triphosphate, Sonogashira coupling, phosphoromorpholidate, phosphorylation, primer extension

## Abstract

A general approach is presented for synthesizing alkyne-modified nucleoside triphosphates via the Sonogashira cross-coupling reaction of unprotected halogenated 2ʹ-deoxynucleoside, followed by monophosphorylation and the reaction of the corresponding phosphoromorpholidate with tributylammonium pyrophosphate. A highly efficient approach for the milligram-scale synthesis of base-modified nucleoside triphosphates with an amino acid-like side chain was developed. The present chemical method outweighs the other reported methods of a base-modified nucleoside triphosphates synthesis in terms of it being a protection-free strategy, the shortening of reaction steps, and increased yields (about 70%). The resulting 8-alkynylated dATP was tested as a substrate for DNA polymerases in a primer extension reaction.

## 1. Introduction

The synthesis of new nucleotide analogs has been prompted by a variety of applications of chemically modified nucleotides, including anticancer and antiviral drugs, biochemical probes, anti-Parkinson’s agents, and bacterial inhibitors [1,2,3]. Introducing functional groups, which resemble amino acid side chains or fragments of small molecules, at the DNA nucleobase enhances the ligand-binding properties and SELEX efficiency [4,5,6,7]. Among the large number of publications related to modified nucleotides, researchers have predominantly focused on modifying the C5 position of pyrimidines [8,9,10,11] and the C6 position of purine nucleosides [12,13,14], while a substitution at the C8 position [15,16,17] has been minimally explored.

Hydrophobic functional groups are the most effective modifications for a wide range of protein targets using the SELEX methodology [5,6,7]. Therefore, the model compound chosen in this study was 8-alkynyl-2ʹ-deoxyadenosine-5′-triphosphate (**6**). In this strategy, the hydrophobic aromatic side chain is directly attached to the C8 position of the purine moiety by a small, linear, and rigid alkyne connection. C8-Alkyne purine derivatives cause a minor decrease in the melting temperature of dsDNA and do not disturb the helical structures [4].

The alkynylation of nucleosides is a crucial modification technique [18,19,20]. The Sonogashira coupling of alkynes with unprotected halogenated nucleosides, catalyzed using a water-soluble palladium/copper catalyst system, is an effective method of introducing substituents through newly formed carbon–carbon bonds [10,21,22]. This study reports suitable reaction conditions for the selective 5′-phosphorylation of unprotected 8-alkynyl-2′-deoxyadenosine through the formation of a corresponding phosphoromorpholidate derivative, resulting in a shorter reaction time and a higher yield. The proposed synthetic protocol is operationally simple, does not require an inert atmosphere, low temperature, or any special precautions, and uses only commercially available reagents. The 5′-selective phosphorylation of nucleosides depends strongly on the nature of the base substituent and the final yield of the base-modified 2′-deoxyadenosine-5′-triphosphate (dNTP) often does not exceed 15%. The synthetic method reported herein provides fast and reliable access to nucleoside triphosphates (NTPs), an amino acid-like side chain holding nucleotides for DNA modification using a primer extension reaction.

## 2. Results and Discussion

### 2.1. Synthesis of 8-Alkyne-Modified 2′-Deoxyadenosine Triphosphate

**Scheme 1:**
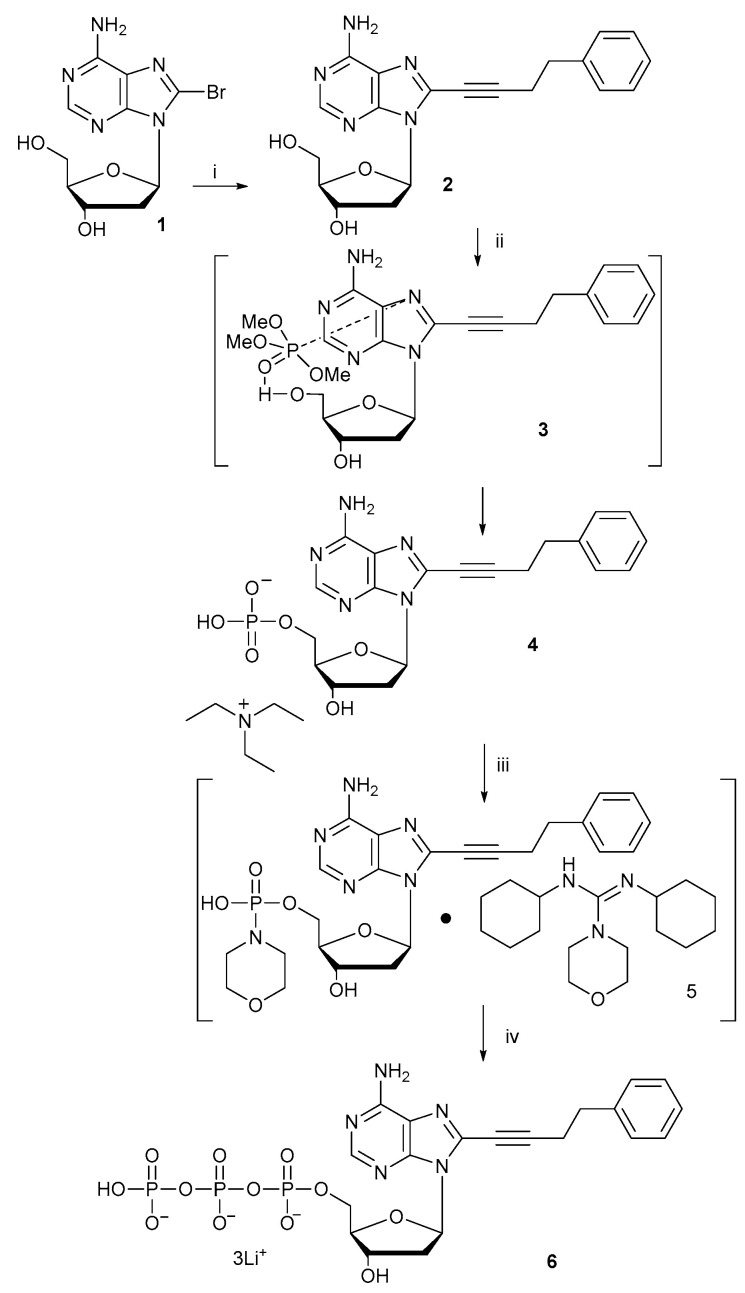
Reagents and conditions: i, 4-phenyl-1-butyne, CuI, Pd(OAc)_2_, TPPTS, DMF, DIPEA, 20 °C, 24 h, 74%; ii, 1. POCl_3_, (MeO)_3_PO, 4 °C, 10 min; 2.1 M triethylammonium bicarbonate buffer (TEAB), 20 °C; iii, morpholine, DCC, hexamethylphosphortriamide (HMP), 70 °C, 1.5 h, 70%; iv, (NBu_4_)_3_HP_2_O_7_, 4,5-dicyanoimidazole (DCI), HMP, 35 °C, 25 h, 64%.

The revised method for synthesizing modified adenosine is outlined in Scheme 1. It involves a Sonogashira cross-coupling reaction of an unprotected halogenated-2′-deoxy nucleoside, followed by monophosphorylation and the reaction of the corresponding phosphoromorpholidate with tributylammonium pyrophosphate. 8-Bromo-2′-deoxyadenosine **1** is readily available from commercial sources or can be obtained through halogenation of the natural nucleoside [15]. A Sonogashira cross-coupling reaction of unprotected 8-bromo-2′-deoxyadenosine **1** and 4-phenyl-1-butyne was performed using Pd(OAc)_2_ and a water-soluble ligand, P(PhSO_3_Na)_3_ (triphenylphosphine-3,3′,3″-trisulfonic acid trisodium salt, TPPTS) [16], in the presence of CuI and *N*,*N*-diisopropylethylamine (DIPEA). Typically [16], the optimal yield of modified adenosine is achieved when using an aqueous solvent (1:1, H_2_O/CH_3_CN). However, 8-bromoadenosine **1** exhibits poor solubility under these conditions. In polar aprotic solvents like DMF [21,22], the reaction proceeded smoothly, resulting in a high yield of the desired product.

It is critical to perform the reaction under oxygen-free conditions to obtain product **2** at a high yield [20]. To achieve this, it is strongly advised to thoroughly deoxygenate the solvent and seal the reaction vessel under an inert atmosphere. When exposed to air in the presence of DIPEA, the copper co-catalyst undergoes homocoupling (Glaser homocoupling), which lowers the efficiency of cross-coupling with the halogenated adenosine [23,24]. This side process results in a significant yield reduction because of the unwanted wastage of the terminal alkyne.

In this study, the freeze–thaw technique proved to be the most effective degassing method for Sonogashira cross-coupling [20]. To simplify the Sonogashira cross-coupling procedure, the reaction mixture containing all reagents except for DIPEA was dissolved in an anhydrous DMF and frozen through the immersion of the reaction vessel in liquid nitrogen. Finally, DIPEA was added to the frozen reaction mixture and pumped it out. By following this protocol, it was possible to prepare the desired product without any significant homodimerization.

Furthermore, the conversion of a nucleoside to its corresponding triphosphate is the most challenging step in NTP synthesis. A wide range of chemical methods [25,26,27,28,29,30] for preparing nucleoside triphosphates rely on the application of activated nucleoside 5′-monophosphates (such as imidazolidate [31,32] or morpholidate [33,34,35]) and a “one-pot, three-step” [36,37,38,39] Kore synthetic strategy starting from the corresponding nucleoside. These methods do not require nucleoside protection, and the reaction proceeds in one pot without isolating any intermediates. The phosphorylation of nucleosides using the Kore method depends strongly on the nature of the substituent in purine bases, with a final yield of the modified dATP of approximately 15–20% [37].

Yoshikawa’s procedure is one of the first and most widely used methods to synthesize nucleoside 5′-triphosphates from their corresponding monophosphates. The procedure selectively phosphorylates unprotected nucleosides at position 5′ [40,41]. However, Yoshikawa’s procedure is not suitable for synthesizing nucleoside monophosphate on a milligram scale, as water dosing problems (1–2 μL) arise. We were unable, however, to reproduce the phosphorylation of 8-bromoadenosine **1** under the Yoshikawa conditions (excess POCl_3_, H_2_O, and triethylphosphate). This method resulted in the production of hydrophobic by-products, and the desired monophosphate **4** was isolated in only a low yield of about 15%.

Based on the Ikemoto optimization of the Yoshikawa reaction [42], 5′ selective phosphorylation of nucleoside with POCl_3_ in trialkyl phosphate proceeds via the formation of the nucleoside–trialkylphosphate complex as the reaction intermediate. In the experiments, the reaction of modified adenosine and trimethyl phosphate at 50 °C for 20 min with subsequent phosphorylation using POCl_3_ at 4 °C led to the formation of nucleoside monophosphate with a 70% yield.

The commonly employed approach for synthesizing nucleoside triphosphates involves converting monophosphates into the appropriate phosphorimidazolides using imidazole and triphenylphosphine/2,2′-dithiodipyridine as the condensing agent in the presence of tributylamine [43]. According to this method, the activated phosphorimidazolide compound reacted with bis(tri-n-butylammonium) pyrophosphate in DMF in the presence of ZnCl_2_ [44]. We attempted to synthesize 8-modified adenosine triphosphate **6** using the same conditions, but a significant amount of hydrophobic by-product was formed, resulting in only trace amounts of the desired triphosphate **6**. Probably, this is due to the high reactivity of the phosphorimidazolide derivative, which results in the formation of the appropriate dinucleoside 5′,5′-diphosphate with a high yield [45,46]. The phosphorimidazolide derivative exhibits a significantly higher reactivity toward various nucleophiles in comparison with the appropriate phosphoromorpholidate [33,34,35].

Therefore, the morpholidate synthetic method was selected for optimization due to its one-pot reaction feature with no need to isolate the reaction intermediate. To optimize the reaction procedure and reduce the formation of by-products, several parameters were modified such as the solvent, time, temperature, activator, and pyrophosphate salt.

The modified dAMP **4** as triethylammonium salt was activated with *N*,*N*′-dicyclohexylcarbodiimide (DCC) in the presence of morpholine, leading to adenosine 5′-phosphoromorpholidate as 4-morpholine *N*,*N*′-dicyclohexylcarboxamide salt **5**. The activation reaction was found to occur most effectively in hexamethylphosphortriamide (HMP). Although other solvents, such as acetonitrile, t-butyl alcohol/water mixture, trimethyl phosphate, and DMSO were examined, none of them provided better results. Only the non-activated by-product was obtained in DMF. Excess DCC was deactivated by adding water to the reaction mixture, followed by evaporation in vacuo. The hydrolysis of adenosine 5′-phosphoromorpholidate 5 produced a small amount of monophosphate (modified dAMP), the only by-product observed. Surprisingly, the addition of a tetrabutylammonium pyrophosphate solution in DMF or DMSO at the second step according to Moffatt’s method [33,34] did not yield modified dATP, even after 7 days. Therefore, an activator, such as 1H-tetrazole [47] or 4,5-dicyanoimidazole (DCI) [48], was added to the adenosine 5′-phosphoromorpholidate and tributylammonium pyrophosphate (Table 1). It appears that tetrazole is a less efficient activator than DCI, and the conversion of phosphoromorpholidate to dATP **6** was very slow (typical reaction times are 7 days).

It was determined that using 20 equivalents of DCI and 10 equivalents of pyrophosphate at 35 °C for 25 h was optimal for achieving a high yield of desired dATP **6** and minimizing the formation of by-products. The reaction rate considerably decreased when the amount of DCI was reduced to five equivalents while pyrophosphate was kept constant. The experiments were carried out at 30 °C, 40 °C, and 50 °C. At high temperatures (40 °C and 50 °C), the formation of dAMP (hydrolysis of adenosine 5′-phosphoromorpholidate), dADP (decomposition of dATP), and dinucleoside 5′,5′-polyphosphate was simultaneously observed. The use of DMSO instead of hexamethylphosphortriamide resulted in the formation of a mixture of modified dATP and dADP at a 1:1 ratio. The optimal temperature for the reaction was 35° C, resulting in a favorable compromise between the reaction time and the product yield.

Pure modified dATP **6** was obtained with a moderate yield using a two-step procedure consisting of DEAE anion exchange chromatography followed by reverse-phase C18-RP chromatography. Thin-layer chromatography revealed the formation of a single major product in all cases. The structures of all synthesized compounds were confirmed using MALDI mass-spectrometry, ^1^H-NMR, ^13^C-NMR, and ^31^P-NMR analyses (Figures S1–S7 in Supplementary Materials).

**Figure 1:**
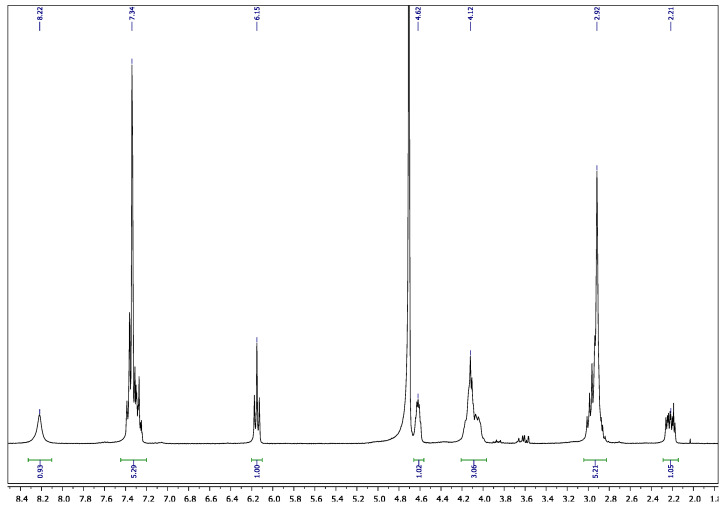
^1^H NMR spectrum of 8-[4-phenyl-but-1-yne-1-yl]-2′-deoxyadenosine-5′-triphosphate trilithium salt (**6**).

NMR spectroscopy is a useful tool for determining structural information about nucleosides. The 2′-deoxyribose sugar can adopt a number of conformations, and these can be an indicator of how similar the modified nucleoside will be to the natural nucleoside in biological systems. The ^1^H-NMR spectra of 2′-deoxyadenosines were expected to display a coupling between all adjacent protons on the sugar ring (an additional coupling to the hydroxyls and amino group was also observed in DMSO-d6) (Figure 1). In addition, the peaks corresponding to the protons of the alkyne linker and phenyl group were present. The ^31^P NMR spectrum of modified dATP showed the triad of signals characteristic for triphosphate (Figure S6).

### 2.2. Enzymatic Incorporation of 8-Alkyne-Modified 2′-Deoxyadenosine Triphosphate during Primer Extensions

The incorporation assays of the modified dATP **6** were attempted with commonly used DNA polymerases from the A and B families, in particular family A polymerases from *Thermus aquaticus* (Taq) [20]. Among the family B DNA polymerases, the ones from *Thermococcus litoralis* [Vent (exo-)], *Pyrococcus* species GB-D [Deep Vent (exo-)], and *Thermococcus kodakaraensis* (Kod XL) were used [4,49,50,51,52]. Primer extensions were performed using two templates.

T1: (T)_20_-TTG-TCA-CTC-AGA-CCA-ACT-CCC-T,
T2.AGCAGCACAGAGG**T**CAGA**T**GCCGCCAGGCCACCCA**T**ACACCAACAACCCCTATGCGTGCTACCGT

**Figure 2:**
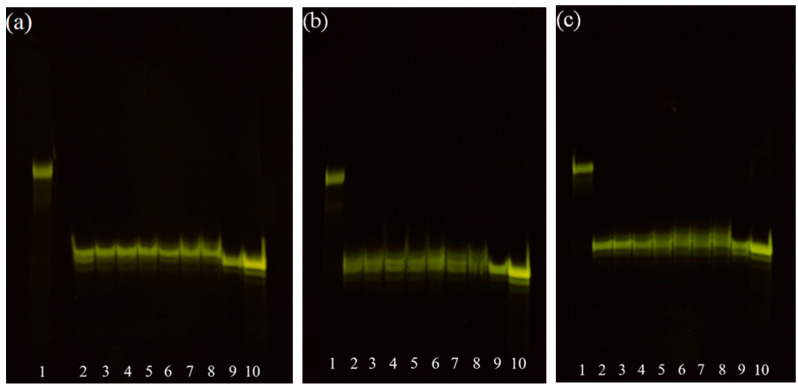
Electrophoretic separation of products of primer extension reaction with 8-modified dATP **6** when reacted with Vent (exo-), Deep Vent (exo-), and Kod XL polymerases (**a**–**c**), respectively, using the T1 template for 5 (lane 2), 15 min (lane 3), and 30 min (lane 4); 1 (lane 5), 2 (lane 6), 3 (lane 7), and 4 h (lane 8). Lane 1—control with dATP, 1 h; lane 9—negative control; lane 10—primer. Denaturing polyacrylamide gel (20%, acrylamide-bisacrylamide ratio, 19:1), 58 °C, recording in the Fitc dye fluorescence range.

Figure 2 shows a polymerase from the primer extension of B families using the T1 template. The T1 template makes it possible to evaluate the efficiency of the sequential incorporation of modified dATP **6** into the growing DNA strand during PEX. This template required a total of twenty dATPs to be incorporated. As has been shown previously, family B polymerases are superior to the members of family A, in terms of both effectivity and accepting a more extended repertoire of unnatural nucleotides [7]. Indeed, Taq DNA polymerase did not accept 8-substituted dATP as the substrate in the discussed experiments, as no primer extension to the product was observed. With an increase in the incubation time from 5 min to 4 h, the number of incorporated nucleotides and, accordingly, the maximum length of the completed primer, did not change significantly. Unfortunately, no band corresponding to the fully extended product could be identified. In the case of the selected DNA polymerase, the elongation was inhibited when one modified adenosine was present in the template. Although these enzymes accepted the modified nucleotide, Vent (exo-) seems to be superior to KOD XL and Deep Vent (exo-).

**Figure 3:**
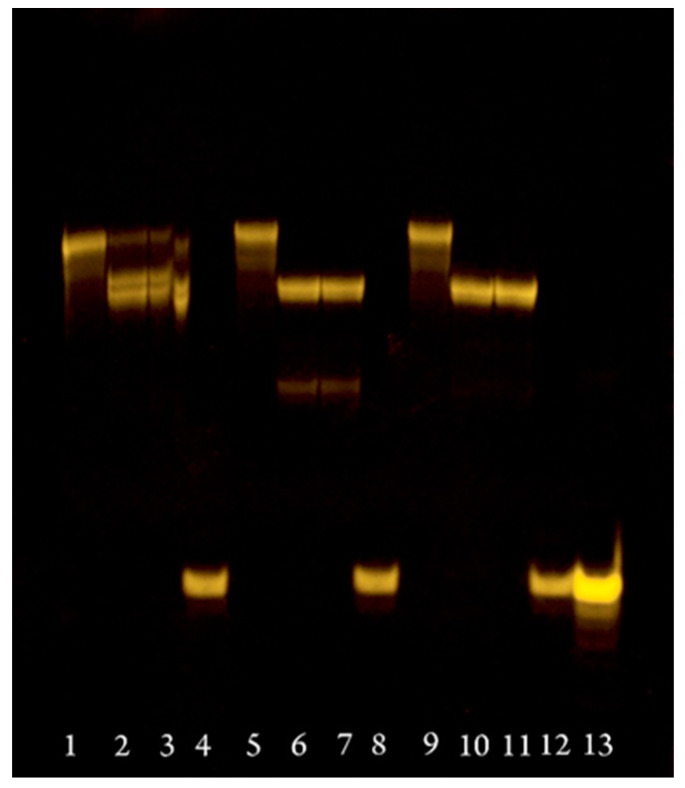
Electrophoretic separation of products of primer extension reaction with 8-modified dATP **6** when reacted with Kod XL, Vent (exo-), and Deep Vent (exo-) polymerases, respectively, using the T2 template, 1 h. Denaturing polyacrylamide gel (20%, acrylamide-bisacrylamide ratio, 19:1), 58 °C, recording in the Cy3 dye fluorescence range. Lanes 1, 5, 9—control with dATP with Kod XL, Vent (exo-), and Deep Vent (exo-), respectively; lanes 2, 3—dATP **6**, Kod XL, two experiments; lanes 6, 7—dATP **6**, Vent (exo-), two experiments; lanes 10, 11—dATP **6**, Deep Vent (exo-), two experiments; lanes 4, 8, 12—negative control; lane 13—primer.

The T2 template is a 66-mer containing a 29 nt long region with three dTTPs separated by sequences containing natural dA, dG, and dC. For the modified dATP **6**, the polymerases of sequence family B were only able to incorporate two modified residues before stopping and showed only a weak elongation of the primer (approximately 47 nucleotides) (Figure 3). The experiments were repeated several times to ensure that the results were reproducible. The band corresponding to the fully extended material was barely visible when using Kod XL polymerase. Based on the data obtained, it follows that in certain cases these polymerases are unable to complete the products after the incorporation of the modified dATP **6**.

## 3. Materials and Methods

### 3.1. General Information

All chemicals were purchased from Sigma-Aldrich (Merck KGaA, Darmstadt, Germany). All chemicals and solvents were of American Chemical Society grade or HPLC purity and were used as received. The ^1^H NMR spectra were recorded with a Bruker AMX-400 spectrometer (400 MHz) (Bruker, Delaware City, DE, USA) in D_2_O and DMSO-d_6_ solutions. Chemical shifts (δ) are given in ppm. Coupling constants (J) are given in Hz. Multiplicities of the signals were as follows: s, singlet; d, doublet; t, triplet; q, quartet; m, multiplet. The mass spectroscopic analysis was performed with a 4800 Plus MALDI-TOF mass spectrometer (Applied Biosystems/MDS Sciex, Foster City, CA, USA). Mass spectra were recorded in the linear mode for positive ions. Adenosine phosphates synthesis was performed using an AccuBlock digital dry bath thermoblock (Labnet International Inc., Edison, NJ, USA). Thin-layer chromatography (TLC) was used as a quality control check of modified adenosine and modified adenosine phosphates to ensure chemical purity. TLC was performed using silica gel 60 RP-18 F254S plates from Merck (Darmstadt, Germany) with mobile phase: acetonitrile/0.1 M TEAHC 20/80 *v*/*v*. Purification of adenosine derivatives was performed using a silica gel (Merck, Darmstadt, Germany), reversed-phase C18-RP (Merck, Germany) and DEAE (Analtech, Newark, DE, USA) column chromatography.

### 3.2. Synthesis of 8-Alkyne-Modified 2′-Deoxyadenosine Triphosphate (***6***)

**8-[4-Phenyl-but-1-yne-1-yl]-2′-deoxyadenosine** (**2**): The Sonogashira coupling reaction was carried out using freeze–pump–thaw degassing methodology. The 4-phenyl-1-butyne (195 mg, 1.5 mmol, 4 equiv.), 8-bromo-2′-deoxyadenosine (**1**) (123 mg, 0.375 mmol), copper (I) iodide (7.1 mg, 38 μmol, 10 mol%), Pd(OAc)_2_ (trimer) (12.5 mg, 19 μmol, 5 mol%), and P(Ph-SO_3_Na)_3_ (27 mg, 47.5 μmol, 2.5 equiv. to Pd) were dissolved in an anhydrous DMF (7.5 mL). The reaction mixture was frozen through immersion of the flask into liquid nitrogen. The frozen flask was pumped out at 0.5 Torr for 5 min while allowing the reaction mixture to defrost. This procedure was repeated three times. Then, DIPEA (654 μL, 10 equiv.) was added to the frozen reaction mixture. The mixture was pumped out and stirred under vacuum at ambient temperature for 24 h in the absence of light. Then, the mixture was diluted with chloroform (30 mL). The products were purified using silica gel column chromatography (32 × 170 mm) using EtOH–CHCl_3_ (5% to 25%) as eluent. The chloroform solution of the modified nucleoside was evaporated in vacuo, the residue was dissolved in CH_3_CN–0.1M TEAB (20%, pH 8.5, 10 mL) and filtered through a short pad of activated Celite^®^ to remove the catalyst traces. Then, water solution was loaded onto a reversed-phase C18-RP column (16 × 300 mm) and eluted using a linear gradient of 35% acetonitrile in 0.1 M TEAB buffer (pH 8.5) to 45% acetonitrile in 0.1 M TEAB buffer (pH 8.5) for 450 min at a flow rate of 0.7 mL/min. The solution was evaporated in vacuo. The crystals formed were dried in a vacuum desiccator over P_2_O_5_. The product was isolated as white powder 105 mg (74%). MS (MALDI-TOF) *m*/*z* calculated for C_20_H_21_N_5_O_3_ 379.42 found 379.59 [M]^+^. ^1^H NMR (DMSO-d6) δ: 2.16 (ddd, J = 6.7, 6.5, 2.5 Hz, 1H, H-2′b), 2.92 (t, J = 3 Hz, 4H, CH_2_CH_2_), 3.09 (ddd, J = 11.3, 8.0, 6.1 Hz, 1H, H-2′a), 3.52 (dd, J = 11.8, 4.7 Hz, 1H, H-5′b), 3.67 (dd, J = 11.8, 4.2 Hz, 1H, H-5′a), 3.91 (q, J = 4.5 Hz, 1H, H-4′), 4.50 (m, 1H, H-3′), 5.34 (br.s., 2H, OH-5’, OH-3’), 6.36 (t, J = 7 Hz, 1H, H-1’), 7.24 (m, 1H, H-*para*-Ph), 7.35 (d, J = 4.5 Hz, 4H, H-*ortho*-Ph, H-*meta*-Ph), 7.49 (br.s., 2H, NH_2_), 8.14 (s, 1H, H-2). ^13^C NMR (DMSO-d6) δ: 155.87, 152.96, 148.25, 139.86, 133.25, 128.42, 128.33, 126.31, 119.09, 96.94, 88.26, 85.02, 71.21, 70.61, 62.20, 37.60, 33.33, 20.58.

**8-[4-Phenyl-but-1-yne-1-yl]-2′-deoxyadenosine-5′-monophosphate triethylammonium salt** (**4**)**:** A suspension of modified adenosine **2** (10 mg, 26.4 μmol) in trimethyl phosphate (150 μL) and tributylamine (14 μL, 59.1 μmol) was heated at 50 °C for 20 min, then cooled to 20 °C for 15 min and to 4 °C for additional 30 min. The phosphorous oxychloride (7.5 μL, 79 μmol) in trimethyl phosphate (92 μL) was added at once under cooling at 4 °C to the reaction mixture and further stirred for 10 min. The reaction mixture was then quenched through the slow addition of 0.2 M triethylammonium bicarbonate buffer (TEAB, pH 8.5, 1 mL) under cooling to 4 °C for 1 h, diluted to 25 mL with CH_3_CN–0.1M TEAB (5%, pH 8.5, 25 mL), loaded onto a reversed-phase C18-RP column (16 × 300 mm), and eluted using a linear gradient of 5% acetonitrile in 0.05 M TEAB buffer (pH 8.5) to 30% acetonitrile in 0.05 M TEAB buffer (pH 8.5) for 300 min at a flow rate of 0.8 mL/min. The fraction containing the modified dAMP **4** was pooled and lyophilized overnight. The crystals formed were dried in a vacuum desiccator over P_2_O_5_. The product was isolated as white powder 7 mg (70%). MS (MALDI-TOF) *m*/*z* calculated for C_20_H_23_N_5_O_6_P 539.38 found 538.51 [M]^−^. ^1^H NMR (D_2_O) δ: 2.19 (ddd, J = 11.1, 7.4, 4.5 Hz, 1H, H-2′b), 2.84 (m, 4H, CH_2_CH_2_), 3.04 (m, 1H, H-2′a), 3.82 (m, 1H, H-5′b), 3.89 (m, 1H, H-5′a), 4.02 (m, 1H, H-4′), 4.57 (m, 1H, H-3′), 6.10 (t, J = 7.0 Hz, 1H, H-1′), 7.24 (m, 5H, H-Ph), 8.07 (s, 1H, H-2). ^31^P NMR (D_2_O) δ: 4.03 (s, P_α_). ^13^C NMR (DMSO-d6) δ: 154.73, 153.20, 148.14, 140.16, 134.75, 128.63, 128.54, 126.64, 118.24, 99.86, 85.51, 85.41, 83.83, 71.22, 69.63, 63.89, 63.83, 36.07, 32.91, 20.72.

**8-[4-Phenyl-but-1-yne-1-yl]-2′-deoxyadenosine-5′-triphosphate trilithium salt** (**6**)**:** The modified adenosine monophosphates as triethylammonium salt **4** (5.3 mg, 9.3 μmol), morpholine (20 μL, 0.232 mmol), and dicyclohexylcarbodiimide (15.2 mg, 74 μmol) were dissolved in an hexamethylphosphortriamide (300 μL) at 70 °C for 1.5 h. The morpholine from the reaction mixture was evaporated at 0.5 Torr at 50 °C. The residue was diluted with Milli-Q water (1 mL) under cooling at 5 °C and then water was evaporated at 0.5 Torr at 40 °C for 40 min. This procedure was repeated twice. Then, tris(tetrabutylammonium) hydrogen pyrophosphate (84 mg, 93 μmol), 4,5-dicyanoimidazole (22.3 mg, 186 μmol), and hexamethylphosphortriamide (600 μL) were added to the reaction mixture and stirred at 35 °C for 25 h. The modified triphosphate **6** was separated from 4,5-dicyanoimidazole using portioned precipitation with a 2% solution of LiClO_4_ in acetone. The precipitates were collected using centrifugation at 13,000 rpm for 3 min, washed with acetone (1.5 mL), dissolved in 10 mL of 0.1 M TEAB buffer (pH 8.5), loaded onto a reversed-phase C18-RP column (16 × 300 mm), and eluted using a linear gradient of 2% acetonitrile in 0.1 M TEAB buffer (pH 8.5) to 10% acetonitrile in 0.1 M TEAB buffer (pH 8.5) for 600 min at a flow rate of 1 mL/min. The fraction containing the modified nucleoside triphosphate was concentrated in vacuo, diluted to 15 mL with 30% acetonitrile in Milli-Q water, and loaded onto DEAE-cellulose (DE-52, Whatman) column (17 × 80 mm) equilibrated with 30% acetonitrile in Milli-Q water. Elution was carried out with salt gradient concentration running from 30% acetonitrile in 0.025M TEAB buffer (pH 8.5) to 30% acetonitrile in 0.5M TEAB buffer (pH 8.5) for 300 min at a flow rate of 0.8 mL/min. The solution was evaporated in vacuo. The product was precipitated with a 2% solution of LiClO_4_ in acetone and centrifugated at 13,000 rpm for 2 min, washed with acetone (1 mL), and dried in a vacuum desiccator over P_2_O_5_. The product was isolated as white powder 4 mg (64%). MS (MALDI-TOF) *m*/*z* calculated for C_20_H_24_N_5_O_12_P_3_ 619.36 found 618.44 [M]^−^. ^1^H NMR (D_2_O) δ: 2.21 (ddd, J = 11, 7.5, 4.5 Hz, 1H, H-2′b), 2.92 (m, 5H, CH_2_CH_2_, H-2’a), 4.12 (m, 3H, H-5’b, H-5’a, H-4’), 4.62 (m, 1H, H-3’), 6.15 (t, J = 7 Hz, 1H, H-1’), 7.34 (5H, H-Ph), 8.22 (s, 1H, H-2). ^31^P NMR (D_2_O) δ: -22.61 (s, P_β_), -11.01 (s, P_α_, P_γ_). ^13^C NMR (DMSO-d6) δ: 152.89, 150.40, 148.12, 140.35, 135.65, 128.81, 126.82, 118.09, 100.62, 85.29, 85.19, 84.39, 70.89, 69.68, 65.47, 36.47, 32.91, 20.87.

### 3.3. Enzymatic Incorporation of Modified dATP 6 during Primer Extensions

The reaction of primer extension was carried out using template T1 or T2 and primer P1 or P2 (5’-3’ sequences are shown):

T1: (T)_20_-TTG-TCA-CTC-AGA-CCA-ACT-CCC-T;
Primer1: Fitc-A-GGG-AGT-TGG-TCT-GAG-TGA-CAA;
T2: AGCAGCACAGAGG**T**CAGA**T**GCCGCCAGGCCACCCA**T**ACACCAACAACCCCTATGCGTGCTACCGT;
Primer2: Cy3-ACG-GTA-GCA-CGC-ATA-GG.

The reaction mixtures used for primer extension (25 μL) contained 5 μM primer, 5 μM template, dTTP, dCTP, dGTP, and dATP (or modified dATP **6**) at a concentration of 200 μM and 1 units of Vent (exo-), Deep Vent (exo-), or KOD XL in the enzyme reaction buffer (10×, 2.5 μL) as supplied by the manufacturer and 2.5 mM MgSO_4_ (as case Vent (exo-) and Deep Vent (exo-)). Primer extension was performed through an initial denaturing at 95 °C for 5 min, followed by the elongation time from 5 min to 4 h at 72 °C. Reactions were terminated by adding 25 μL of 0.5 M EDTA, pH 8.0, into each of the tube. The reaction products were isolated and analyzed using electrophoresis under denaturing conditions.

## 4. Conclusions

A highly efficient approach for the synthesis of the alkyne-modified nucleoside triphosphates from the unprotected halogenated-2′-deoxy-nucleoside using palladium-catalyzed cross-coupling chemistry with subsequent selective 5′-phosphorylation was developed. The present method outweighs the other reported methods in terms of being a protection-free strategy, the shortening of reaction steps, and the increased yields, which allows the efficient milligram-scale synthesis of nucleoside triphosphates of an amino acid-like side chain holding nucleotides in good yields with high purity, which would be difficult to prepare using other methods. The synthetic method reported herein provides fast and reliable access to modified nucleoside triphosphates, an amino acid-like side chain holding nucleotides for DNA modification. The resulting 8-alkynylated dATP was further tested as a substrate for DNA polymerases in a primer extension reaction. This study found that 8-alkyne-modified dATP could not be readily incorporated using commonly used polymerases from the A and B families. It is essential to report these findings to characterize the current limitations of enzymatic incorporation for those pursuing the use of 8-modified dATPs as a substrate for DNA polymerases.

## Figures and Tables

**Table 1:** Optimization of the formation of modified dATP from the appropriate phosphoromorpholidate.

Activator, eqv.		(nBu_4_N)_3_HP_2_O_7_, eqv.	Solvent	t, °C	Time	Yield
-		3	DMF, DMSO	20	7 days	traces
				40	2 days	mixture of dAMP, dADP and dATP
1H-tetrazole	4	2	DMF, DMSO, HMP	20	7 days	10%
				50	1 day	mixture of dAMP, dADP and dATP
4,5-dicyanoimidazole	4	1	DMF	20	6 h	19%
				40		mixture of dAMP, dADP and dATP
	20	10	HMP	30	25 h	51%
				35		64%
				40	7 h	dAMP, dADP, dATP, dinucleoside 5′,5′-polyphosphate
				50	6 h	
			DMSO	40	6 h	dATP/dADP, 1:1 ratio
	15	5	HMP	35	25 h	25%
	10	5				19%
	5	10				13%

## Data Availability

Data are contained within the article and Supplementary Materials.

## Supplementary Materials

The following supporting information can be downloaded at: https://www.mdpi.com/article/10.3390/molecules29194747/s1, ^1^H-NMR, ^13^C-NMR and ^31^P NMR of synthesized compounds.
