# Supplementary material for: Optimized Method for the Synthesis of Alkyne-Modified 2′-Deoxynucleoside Triphosphates
Source: Molecules. 2024 Oct 8;29(19):4747. doi: 10.3390/molecules29194747 (PMC11477703; doi:10.3390/molecules29194747)
Supplement: Supplementary file 1 [file molecules-29-04747-s001.zip › molecules-3231246-supplementary.pdf]

SUPPLEMENTARY MATERIALS

New Optimized Method for the Synthesis of Alkyne-Modified 2′-Deoxynucleoside Triphosphates

Viktoriya E. Kuznetsova, Valeriy E. Shershov, Georgiy F. Shtylev, Ivan Yu. Shishkin, Veronika I. Butvilovskaya, Andrey A. Stomakhin, Irina V. Grechishnikova, Olga A. Zasedateleva, and Alexander V. Chudinov**

Engelhardt Institute of Molecular Biology, Russian Academy of Sciences, 119991 Moscow, Russia.

**E-mail:* kuzneimb@gmail.com; chud@eimb.ru.

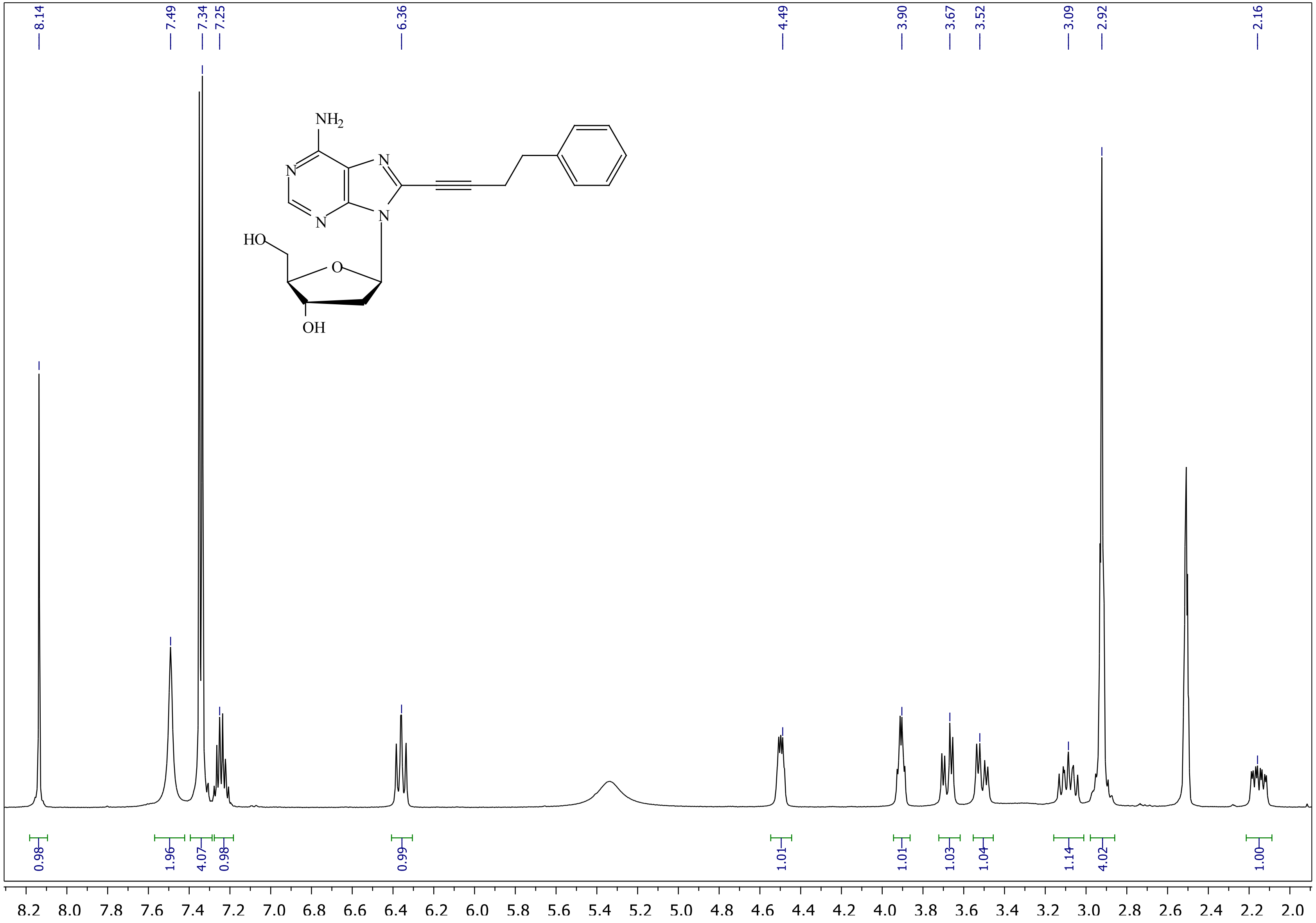

Fig. S1. ^{1}H NMR spectrum of 8-[4-phenyl-but-1-yne-1-yl]-2′-deoadenosine (**2**).

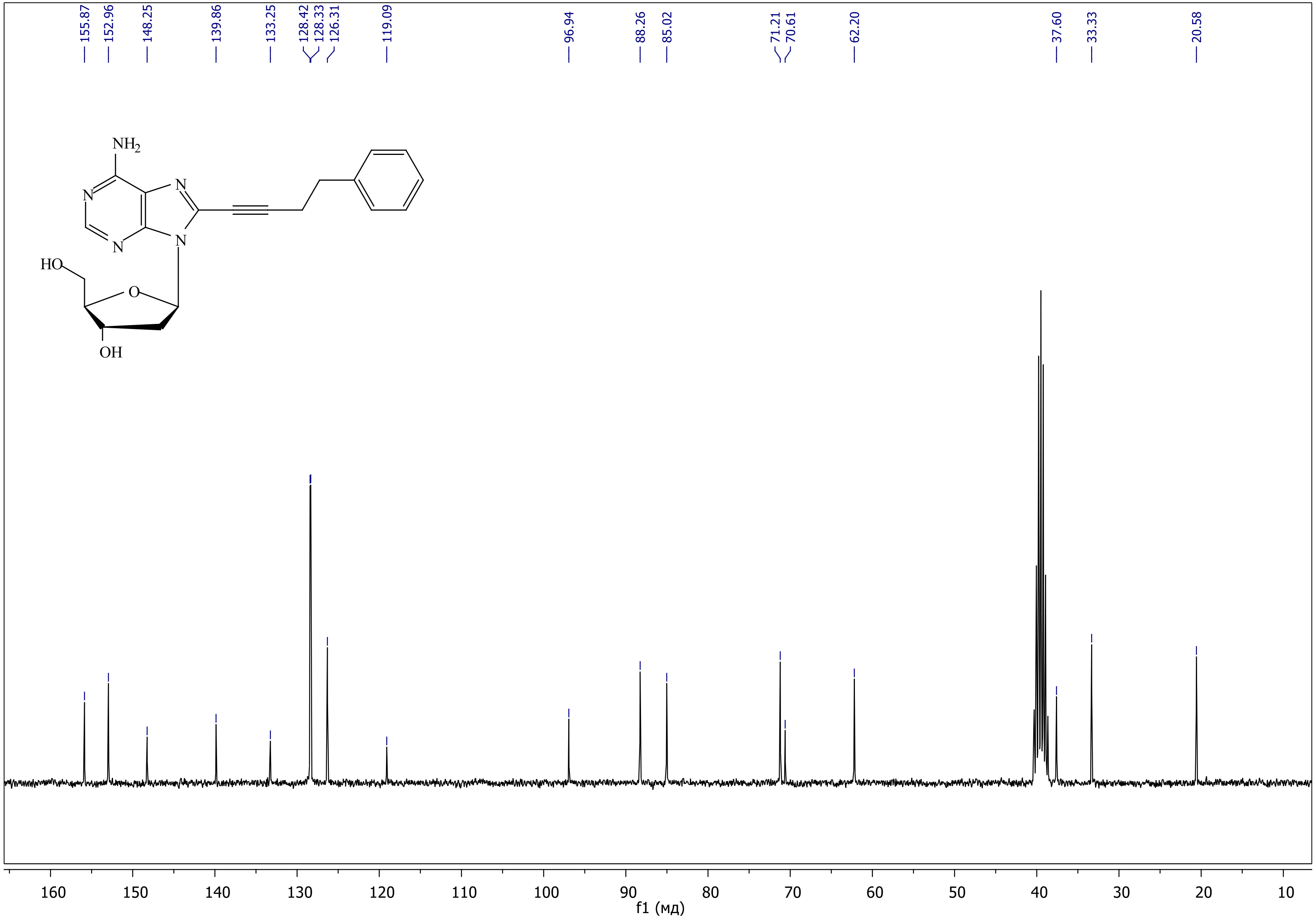

Fig. S2. ^{13}C NMR spectrum of 8-[4-phenyl-but-1-yne-1-yl]-2′-deoadenosine (**2**).

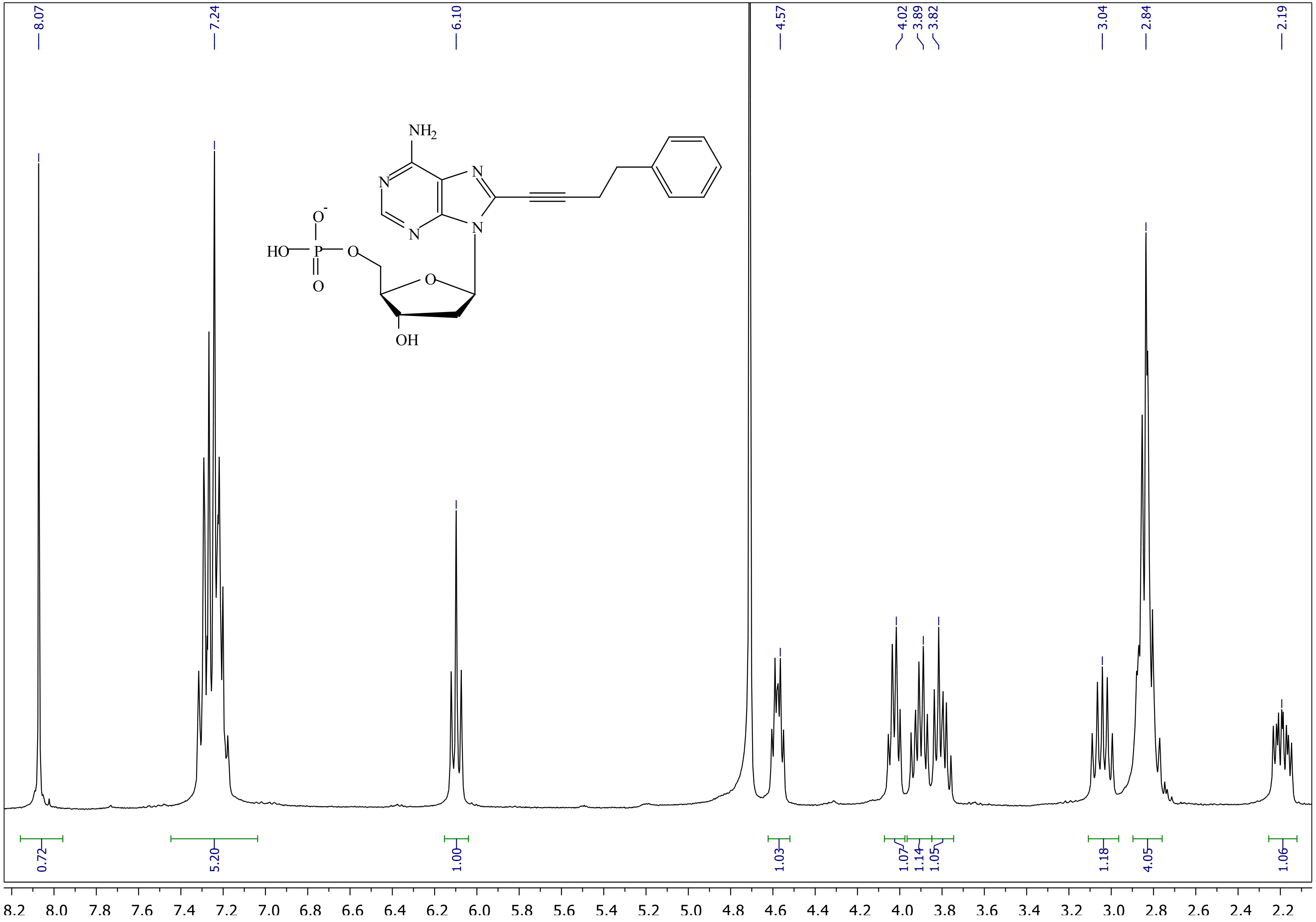

Fig. S3. ^{1}H NMR spectrum of 8-[4-phenyl-but-1-yne-1-yl]-2′-deoadenosine-5′-monophosphate lithium salt (**4**).

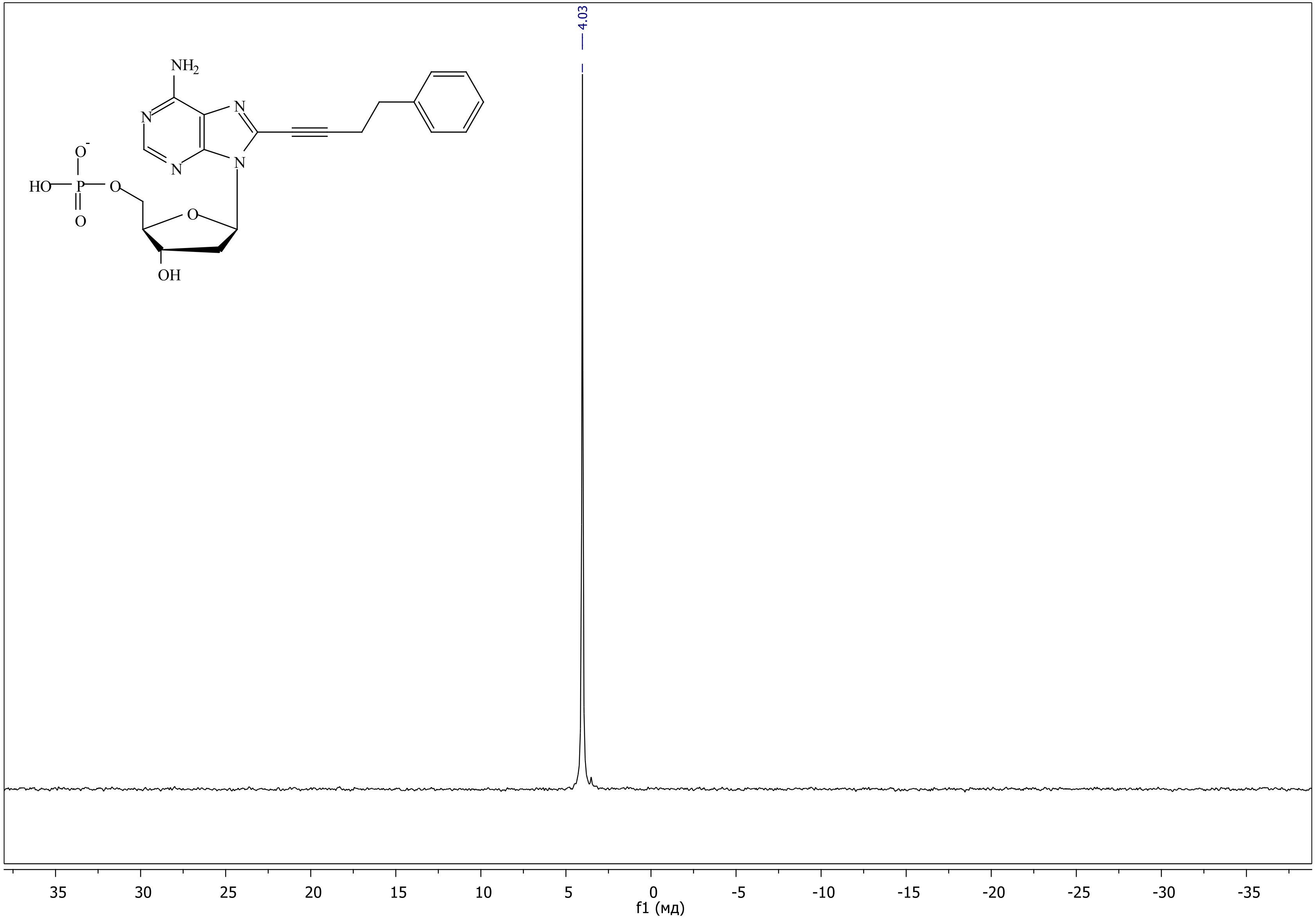

Fig. S4. ^{32}P NMR spectrum of 8-[4-phenyl-but-1-yne-1-yl]-2′-deoadenosine-5′-monophosphate lithium salt (**4**).

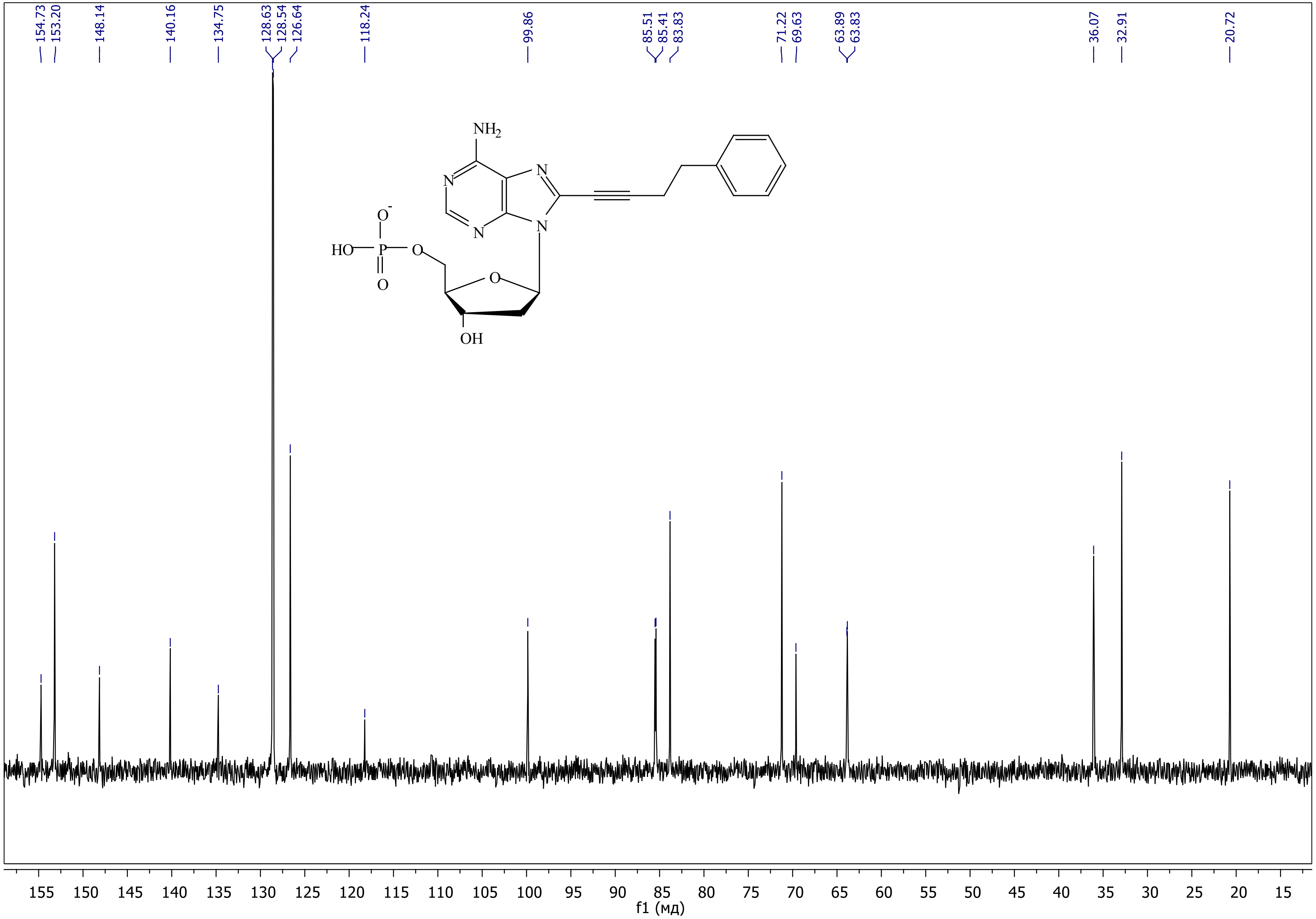

Fig. S5. ^{13}C NMR spectrum of 8-[4-phenyl-but-1-yne-1-yl]-2′-deoadenosine-5′-monophosphate lithium salt (**4**).

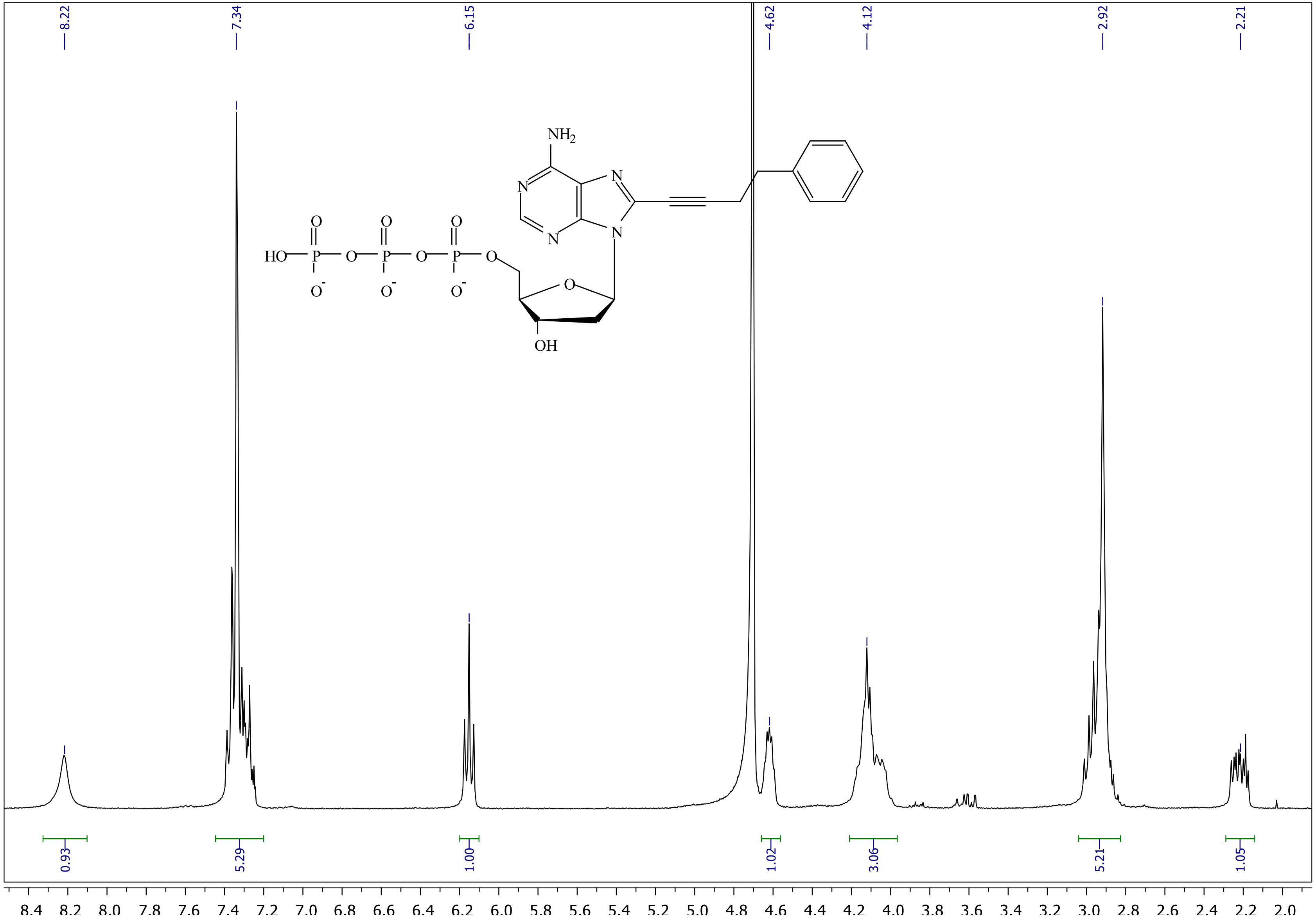

Fig. S6. ^{1}H NMR spectrum of 8-[4-phenyl-but-1-yne-1-yl]-2′-deoadenosine-5′-triphosphate trilithium salt (**6**).

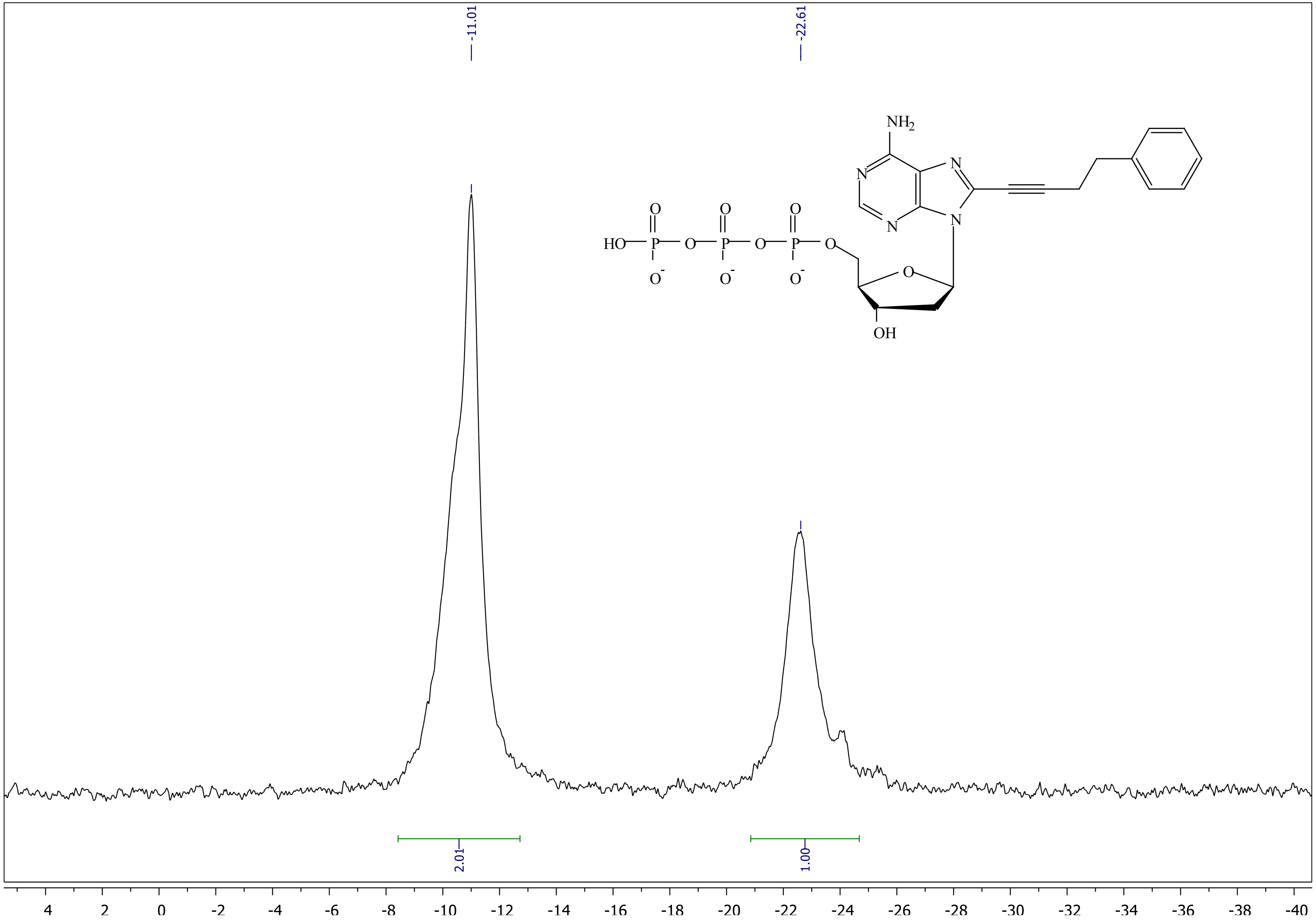

Fig. S7. ^{32}P NMR spectrum of 8-[4-phenyl-but-1-yne-1-yl]-2′-deoadenosine-5′-triphosphate trilithium salt (**6**).

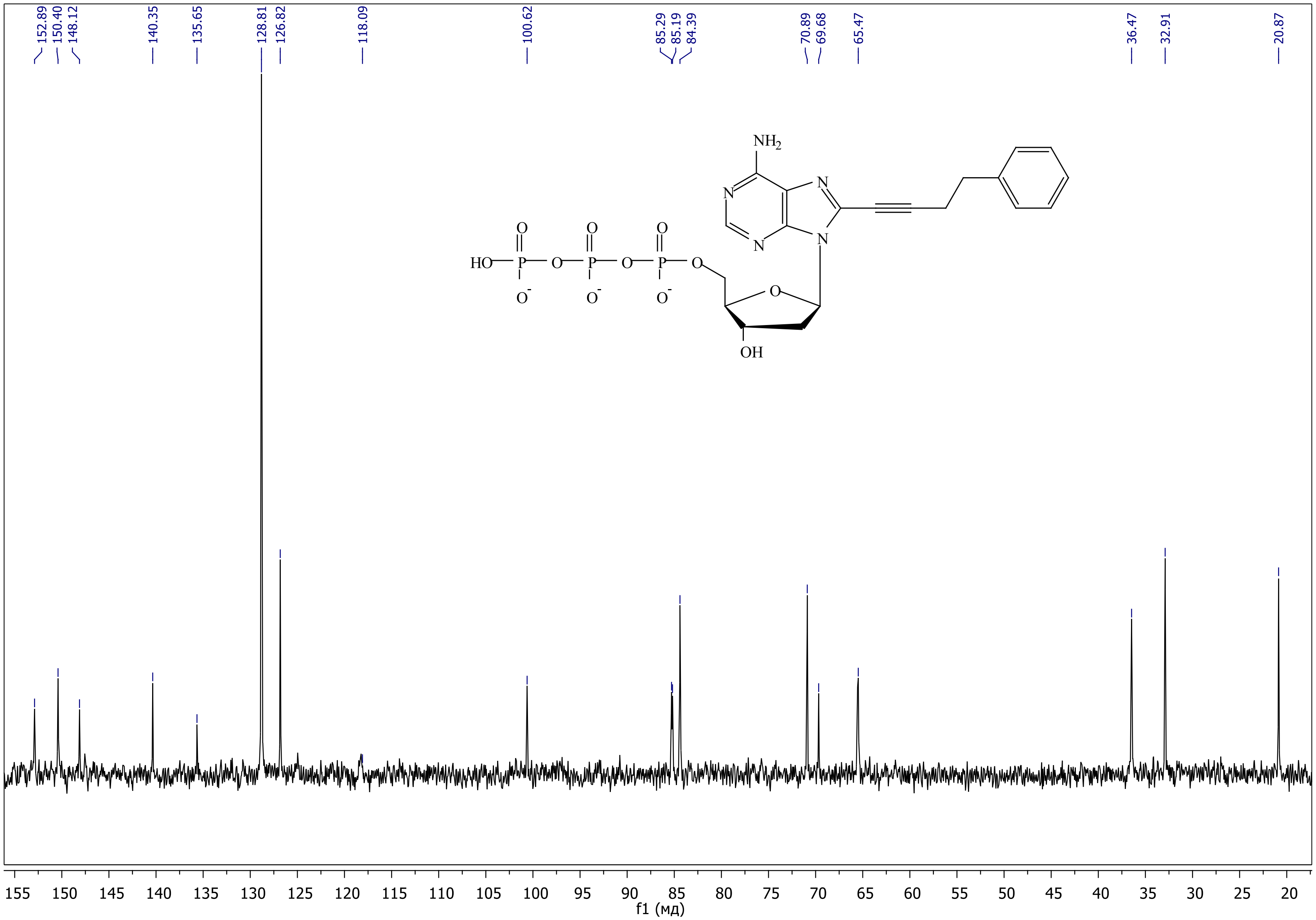

Fig. S8. ^{13}C NMR spectrum of 8-[4-phenyl-but-1-yne-1-yl]-2′-deoadenosine-5′-triphosphate trilithium salt (**6**).
